# ^68^Ga-PSMA-PET/CT and Diffusion MRI Targeting for Cone-Beam CT-Guided Bone Biopsies of Castration-Resistant Prostate Cancer Patients

**DOI:** 10.1007/s00270-019-02312-8

**Published:** 2019-08-23

**Authors:** T. R. F. van Steenbergen, M. Smits, T. W. J. Scheenen, I. M. van Oort, J. Nagarajah, M. M. Rovers, N. Mehra, J. J. Fütterer

**Affiliations:** 1 Department of Radiology and Nuclear Medicine, Radboud Institute for Health Sciences, Radboud University Medical Center, P.O. Box 9101, 6500 HB Nijmegen, The Netherlands; 2 Department of Medical Oncology, Radboud Institute for Molecular Life Sciences, Radboud University Medical Center, Nijmegen, The Netherlands; 3 Department of Operating Rooms, Radboud Institute for Health Sciences, Radboud University Medical Center, Nijmegen, The Netherlands; 4 Department of Urology, Radboud Institute for Health Sciences, Radboud University Medical Center, Nijmegen, The Netherlands

**Keywords:** Castration resistant prostate cancer, Molecular analysis, Bone biopsy, PSMA-PET/CT, Diffusion MRI, Cone-beam CT

## Abstract

**Introduction:**

Precision medicine expands the treatment options for metastatic castration-resistant prostate cancer (mCRPC) by targeting druggable genetic aberrations. Aberrations can be identified following molecular analysis of metastatic tissue. Bone metastases, commonly present in mCRPC, hinder precision medicine due to a high proportion of biopsies with insufficient tumor cells for next-generation DNA sequencing. We aimed to investigate the feasibility of incorporating advanced target planning and needle guidance in bone biopsies and whether this procedure increases biopsy tumor yield and success rate of molecular analysis as compared to the current standards, utilizing only CT guidance.

**Materials and Methods:**

In a pilot study, ten mCRPC patients received ^68^Ga-prostate-specific membrane antigen (PSMA)-PET/CT and diffusion-weighted MRI as biopsy planning images. These datasets were fused for targeting metastatic lesions with high tumor densities. Biopsies were performed under cone-beam CT (CBCT) guidance. Feasibility of target planning and needle guidance was assessed, and success of molecular analysis and tumor yield were reported.

**Results:**

Fusion target planning and CBCT needle guidance were feasible. Nine out of ten biopsies contained prostate cancer cells, with a median of 39% and 40% tumor cells by two different sequencing techniques. Molecular analysis was successful in eight of ten patients (80%). This exceeds previous reports on CT-guided biopsies that ranged from 33 to 44%. In two patients, important druggable aberrations were found.

**Discussion:**

A biopsy procedure using advanced target planning and needle guidance is feasible and can increase the success rate of molecular analysis in bone metastases, thereby having the potential of improving treatment outcome for patients with mCRPC.

**Level of Evidence:**

Level 4, case series.

## Introduction

Metastatic castration-resistant prostate cancer (mCRPC) is a molecular heterogeneous disease with a high frequency of potentially actionable genetic alterations, supporting use of routine molecular profiling to expand on standard treatment options for these patients [1–3]. Whereas a low tumor yield from tumor biopsies is sufficient for classical pathological diagnostic assessments, a higher tumor content is required for tumor profiling using next-generation sequencing techniques. For targeted or whole genome sequencing (WGS), a minimum input of 50 ng of high-quality DNA is necessary, corresponding to at least 10.000 cells. The preferred location for biopsy is soft tissue, because the tumor yield tends to be higher than in bone [4], and as a consequence, molecular analyses are commonly unsuccessful from bone metastatic sites. In patients with mCRPC, bone is the predominant or sole site of metastases in 80.6% and 42.9% of all cases, respectively [5], leaving these patients underrepresented in molecular studies. Therefore, the medical need exists to collect tissue from bone lesions, to increase the success rate of molecular analyses, particularly from small osteoblastic sites. High tumor yield occurs in sites with high tumor density, which in the case of prostate cancer (PCa) can be detected by ^68^Ga-prostate-specific membrane antigen (PSMA)-PET/CT and diffusion-weighted MRI (DWI) [6, 7]. We hypothesized that incorporating this functional information in biopsy planning using image fusion and displaying the planned path during cone-beam CT (CBCT) guidance can improve the amount of tissue obtained by bone biopsies for molecular characterization.

The aim of this prospective pilot study is to investigate first whether target planning on fused ^68^Ga-PSMA-PET/CT, DWI and CBCT image datasets and CBCT guidance for bone biopsies are feasible and second whether this procedure increases the success rate of molecular analyses on bone metastases in mCRPC patients.

## Materials and Methods

From December 2017 to February 2019, 10 mCRPC patients with at least one bone metastasis on diagnostic imaging and planned molecular analysis were enrolled in this pilot study. Patients with a soft tissue lesion accessible for biopsy were excluded. This study was approved by the local ethical committee. An informed consent was obtained from all participants.

### Metastasis Selection

Patients received both ^68^Ga-PSMA-PET/CT and MRI for diagnostic workup. The nuclear medicine physician identified metastases with the highest uptake, indicating high tumor density. DWI sequences were focused on these metastases. The interventional radiologist then selected an accessible metastasis with low ADC value and high b800 signal intensity as target lesion, because this suggests structural changes by tumor cells.

### Target Planning

**Fig. 1:**
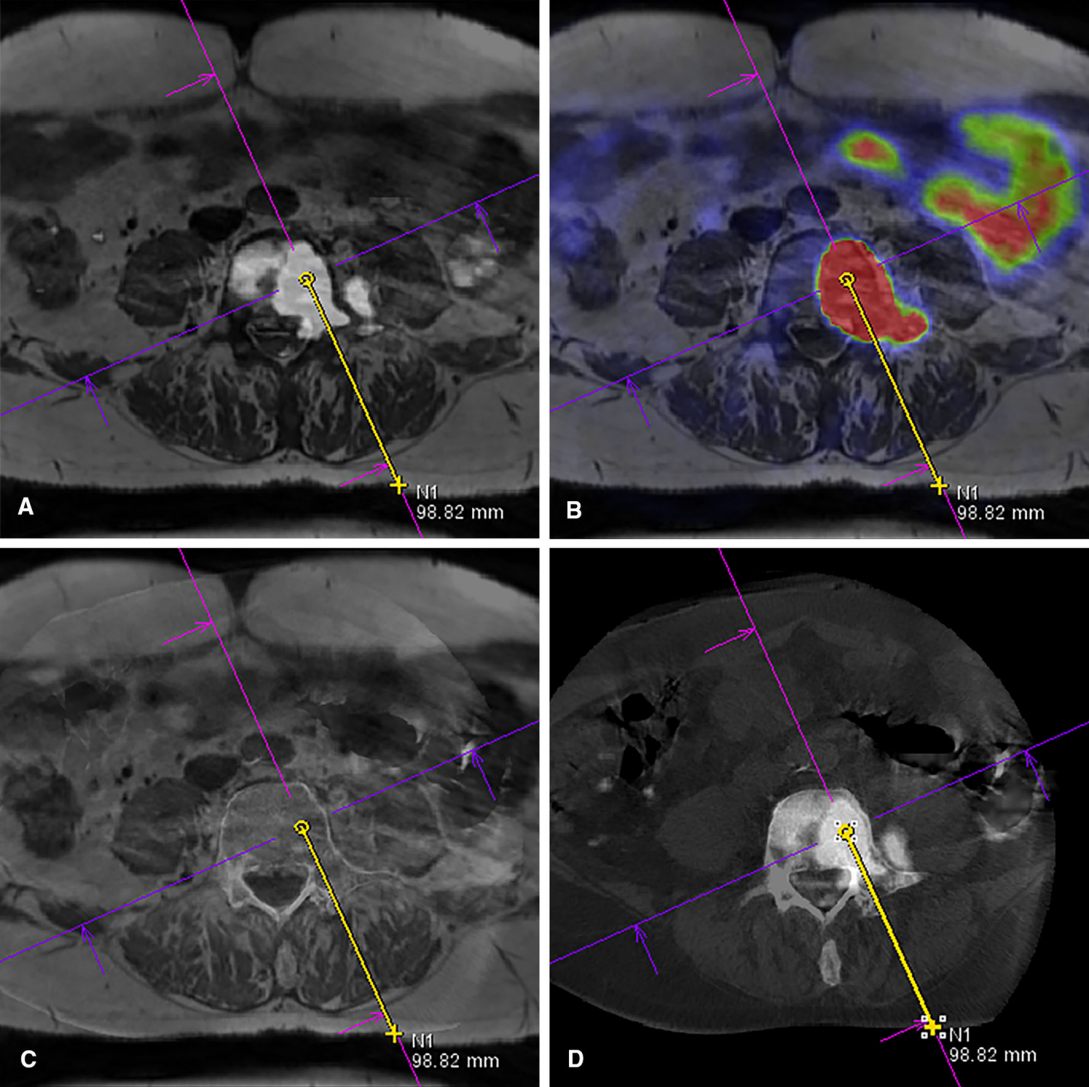
**A**–**D** Target planning of patient 1 on fused datasets. **A** The target was initially planned on a fusion of T1 and b800 images. **B** This target was then verified on a fusion of T1 and ^68^Ga-PSMA-PET images. **C** The CBCT scan showing the current anatomy was fused with the T1 to optimize the needle path. **D** The CBCT/b800 fusion with needle path was displayed on screens in the room during needle guidance

The needle path, a straight line between target and entry point, was planned on a Syngo workstation (Syngo, Siemens Healthineers, Forchheim, Germany). For target planning, the ^68^Ga-PSMA-PET/CT and MRI were fused. Fusion was based on anatomical scans: T1-weighted MRI and low-dose CT (PET/CT), and target point planning was performed on functional scans: DWI with *b* value 800 s/mm^2^ and ^68^Ga-PSMA-PET. The target point was planned on the area with the highest b800 pixel intensity (Fig. 1A) and then verified on PET (Fig. 1B). The skin entry point determined the needle path, balancing between: (1) avoiding vital structures; (2) approaching the bone surface perpendicularly; and (3) avoiding a double oblique angle.

### Biopsy Procedure

**Fig. 2:**
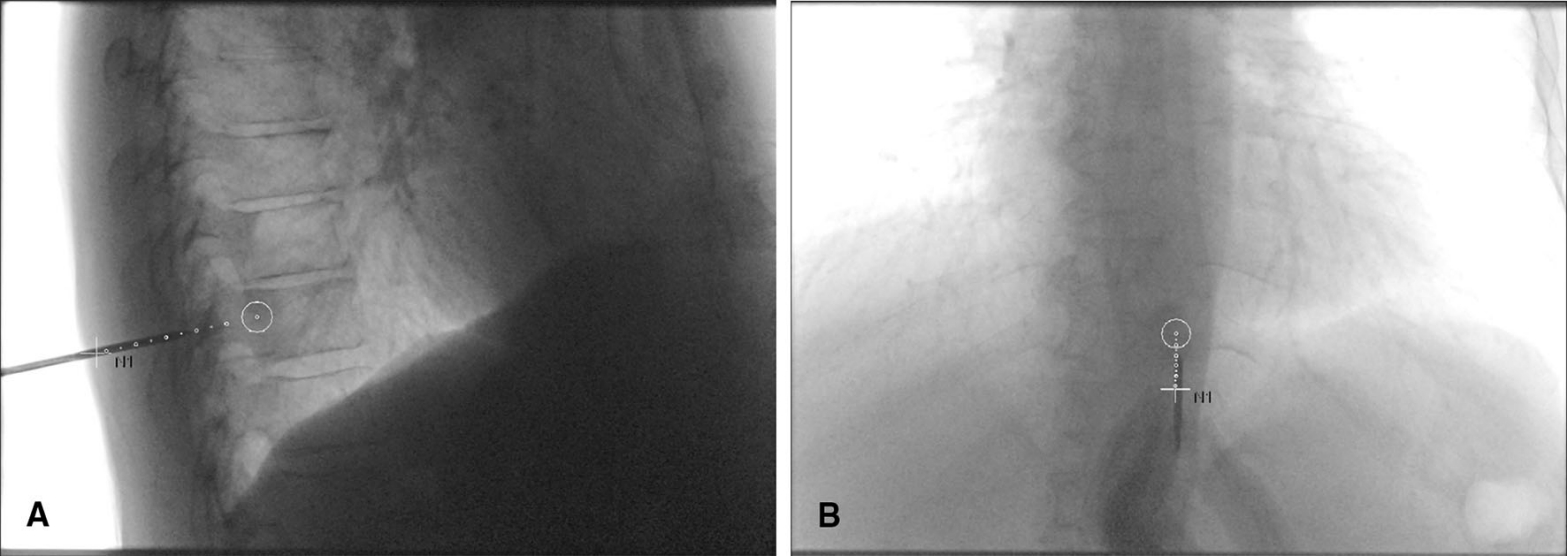
**A**, **B** The planned needle path was overlaid on fluoroscopy images during needle guidance. This example shows the biopsy of the Th11 vertebra in patient 10. Two progression views can be used: **A** a lateral view and **B** a 30° angled frontal view

Patients were prone-positioned on a full carbon table, lying on thin gel mattresses and low pillows as to enable C-arm rotation around the pelvis. Instructions were given to the patients to minimize movement during the procedure, because movement decreases the accuracy of CBCT image fusion. A CBCT was obtained (Artis Zeego, Siemens Healthineers, Forchheim, Germany). The high-dose protocol (6s DynaCT Body, visualization of lower contrast tissue) was used for initial fusion and assessment of vital structures along the needle path (Fig. 1C). Fluoroscopic needle guidance had the needle path overlay in three projection views: one perpendicular to the needle path showing entry point and angle through a laser beam, and two views along the needle path to determine needle progression and deflection in two planes (Fig. 2A, B). After advancing the biopsy needle (Arrow OnControl, Teleflex, Morrisville, NC) to the bone surface, we took a low-dose CBCT (5s DR Body Care, visualization of higher contrast tissue) to verify the needle position in 3D and to correct inaccuracies due to patient movement. If the needle position did not correspond to the planned path, either the needle or planned entry point could be altered. The final needle position was again verified with a low-dose CBCT before taking the biopsy. The window width and level were set to wide and high, respectively, to deal with the needle metal artifacts.

### Outcome Measures

The primary outcome measure was feasibility of the target planning and needle guidance procedures. Target planning was feasible if fusing the scans resulted in precise matching of the target bone and lesion, allowing for target and entry point planning by the operator. Needle guidance was feasible if the needle could be advanced to the target lesion using the fluoroscopy overlay and control CBCT scans, and the interventional radiologist was confident in the final needle position based on a fusion of the final control CBCT and the planning images.

### Molecular Analysis

If the procedure allowed it, a maximum of three 13G biopsy cores were taken. Two molecular analysis techniques were used, each on one core: single molecule molecular inversion probe (smMIP) [8, 9] and whole genome sequencing (WGS) [10]. The second outcome measure was success of analysis and tumor yield, defined as percentage neoplastic cells per biopsy core, reported for both analyzed cores.

## Results

**Fig. 3:**
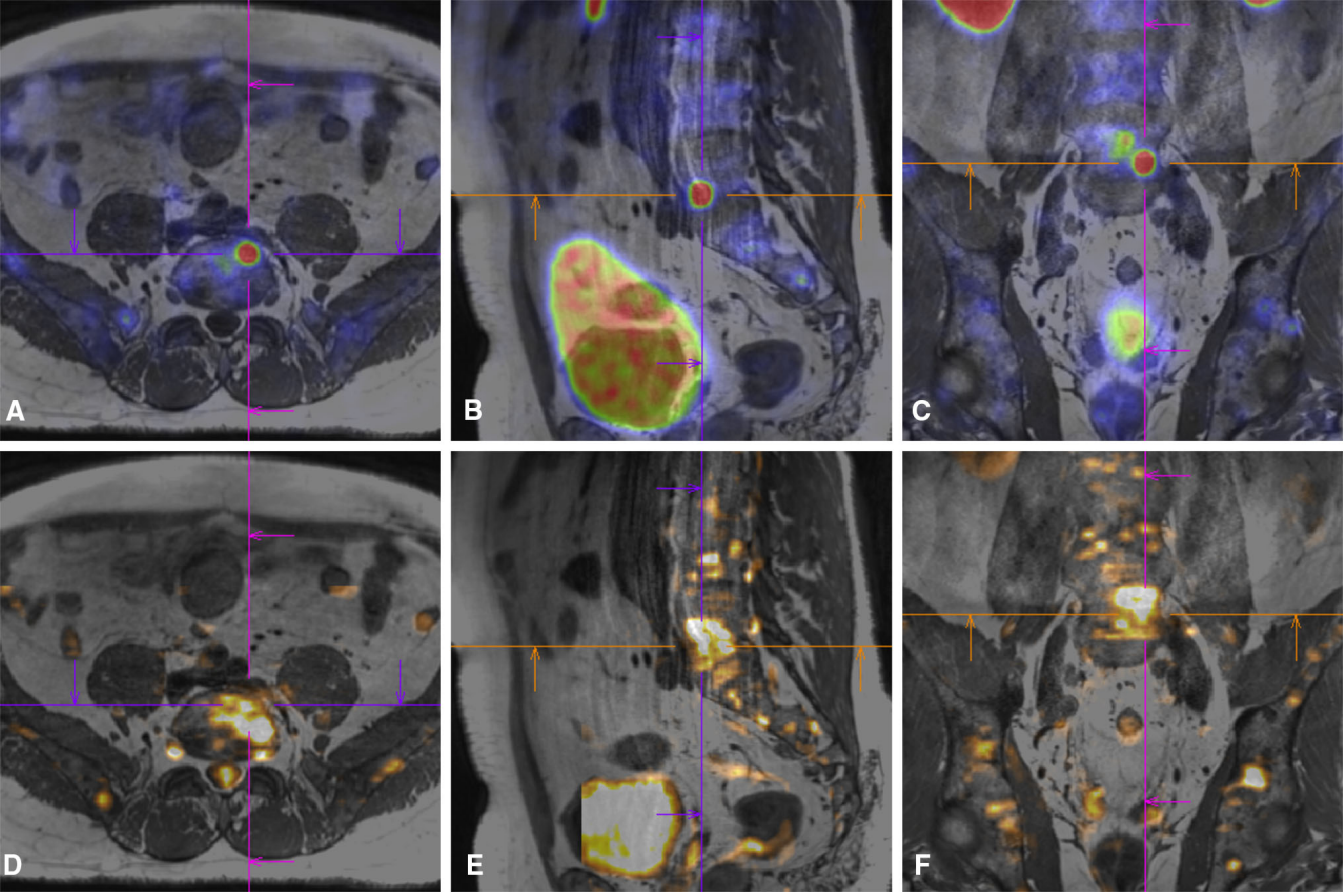
**A**–**F** Image fusion showed the difference in heterogeneity between PSMA uptake and diffusion restriction within some metastases. In this example of patient 2, the lesion in L5 appears larger on DWI than on ^68^Ga-PSMA-PET. **A**–**C**^68^Ga-PSMA-PET and **D**–**F** DWI images are both fused with the T1 MRI, and the lesion is visualized in (**A** + **D**) transversal, (**B** + **E**) sagittal and (**C** + **F**) coronal planes, respectively. The cross is centered at the same position in the T1 MRI for all images and can be used as a reference location. The target point was planned in the regional overlap between high PSMA uptake and high diffusion restriction

**Fig. 4:**
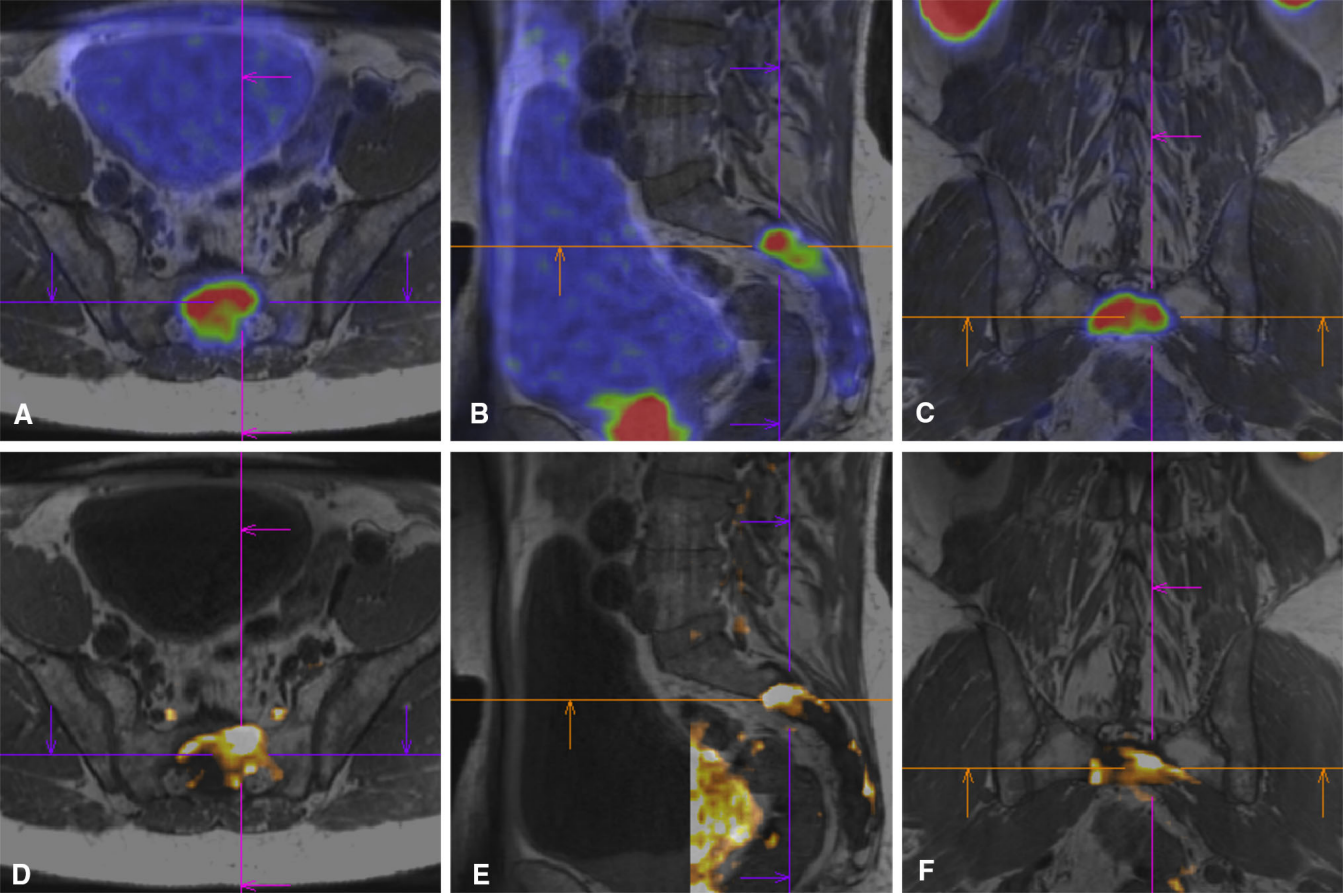
**A**–**F** Image fusion showed the difference in heterogeneity between PSMA uptake and diffusion restriction within some metastases. In this example of patient 8, the lesion in the sacrum appears larger on ^68^Ga-PSMA-PET than on DWI. **A**–**C**^68^Ga-PSMA-PET and **D**–**F** DWI images are both fused with the T1 MRI, and the lesion is visualized in (**A** + **D**) transversal, (**B** + **E**) sagittal and (**C** + **F**) coronal planes, respectively. The cross is centered at the same position in the T1 MRI for all images and can be used as a reference location. The target point was planned in the regional overlap between high PSMA uptake and high diffusion restriction

Fusion target planning and CBCT needle guidance were feasible in all patients. Automatic image fusion did not work in two patients due to an MRI artifact (patient 3) and large difference in spinal curvature (patient 10), necessitating full manual fusion. Minor manual adjustments after automatic fusion were done in six patients, all due to a difference in patient positioning between scans. Image fusion showed the difference in heterogeneity between PSMA uptake and diffusion restriction within some metastases, with regions of high uptake and high restriction not coinciding in three patients. Lesions could appear larger on DWI (Fig. 3) or on ^68^Ga-PSMA-PET (Fig. 4). The target point for these cases was planned on the regional overlap. For some patients, we took more control CBCT scans (Table 1) because following the planned needle path was challenging. This mostly occurred for double oblique needle paths. Ultimately, we preferred the initial CBCT rather than T1-weighted MRI as a base scan for fusion as this greatly sped up fusion with control CBCT scans. Control scans had to be fused manually due to the needle artifacts.

**Table 1:** Patient and procedure characteristics

Patient no.	Age	Location	Number of control CBCT scans	Biopsy contains PCa cells
1.	61	L4	3	Yes
2.	58	L5	4	Yes
3.	71	Os ilium left	2	Yes
4.	57	Os ilium right	1	Yes
5.	62	L1	6	Yes
6.	63	Sacrum	3	Yes
7.	68	Sacrum	2	Yes
8.	64	Sacrum	2	Yes
9.	69	Acetabulum right	1	No
10.	64	Th11	4	Yes

Histopathological examination showed PCa cells in nine of the ten patients (90%) (Table 1). The biopsy cores of one patient did not contain tumor cells because the cortical bone was too compact to take a good biopsy. The nine CRPC-positive biopsies were further analyzed. Tumor yield was > 15% in eight of the smMIP cores (89%). Two cores were eventually not analyzed due to no clinical need, but their tumor yields (15% and 40%) would have sufficed for molecular analysis in the case of good DNA quality. WGS was successful in seven cores (78%). In eight out of ten patients (80%), molecular analysis could be performed by either smMIP or WGSs. The median tumor yield in the WGS cores was 39%, compared to 40% in the smMIP cores. The most identified genetic alterations were loss of PTEN, aberrations in AR, TMRSS2-ERG fusion and mutation in TP53, as expected since these are most commonly identified in CRPC (Table 2). In two patients, we found druggable genetic defects: one patient showed microsatellite instable prostate cancer and could be treated with checkpoint immunotherapy, and one patient had a pathogenic DNA damage repair defect and was eligible for treatment with a poly(ADP-ribose) polymerase inhibitor. In other patients, we identified prognostic aberrations and hotspot mutations in the ligand binding domain of AR, which may lead to promiscuous activation by non-canonical steroid ligands such as prednisolone.

**Table 2:** The tumor yield and mutations found for the single molecule molecular inversion probe (smMIP) analysis and whole genome sequencing (WGS)

Patient no.	smMIP		WGS	
	Pathology tumor yield (%)	Mutations	Molecular tumor yield (%)	Mutations
1.	70–80	No druggable mutations, no MSI	60	AR gain^a^
2.	40	No druggable mutations. Probably loss of MSH2 and MSH6 by immunohistochemistry, indicating MSI^c^	11	Mutation in the ligand binding domain of AR^b^ Tumor mutational load 127
3.	*5*	*Insufficient tumor yield*	*8*	*Insufficient tumor yield*
4.	20–30	Mutation in TP53^a^, no MSI	*0*	*Insufficient tumor yield*
5.	40	Analysis not performed	40	Gain of AR^a^, Fusion of TMPRSS2-ETV4
6.	15	Analysis not performed	65	Mutation in TP53 Gain of AR^b^ Loss of PTEN^a^ and RAD51B^c^ Fusion of TMPRSS2-ERG
7.	30	No druggable mutations, no MSI	22	No alterations
8.	60	No druggable mutations, no MSI	41	Mutation in the ligand binding domain of AR^b^ Fusion of TMPRSS2-ERG
9.	*No tumor*	*No tumor*	*No tumor*	*No tumor*
10.	40	Mutation in TP53^a^, no MSI	39	Mutation in TP53^a^ Loss of PTEN^a^

Italics indicates molecular analysis could not be performed

^a^Aberration with prognostic significance

^b^Aberration with consequences for clinical management

^c^Druggable aberration

## Discussion

Biopsy of bone metastatic lesions for molecular profiling is challenging due to a high proportion of biopsies with insufficient tumor yield. Unguided iliac crest bone marrow biopsies of metastases seen on CT were positive for PCa in 62.5% of the cases, and in only 45% sufficient tumor cells were obtained for molecular analysis [11]. Molecular analysis success rates after CT-guided biopsies from bone tissue were similar, ranging from 33 to 44% of all biopsies [12–14]. This can be compared to the previous performance of our own institute, by looking at the success rate of 103 patients with prostate cancer that we included in the CPCT-02 study, a nationwide study investigating the genomic landscape in relation to response to systemic anticancer therapy using WGS [10]. In these patients, bone biopsies were taken under CT guidance without image fusion, aided by a previously obtained CT scan (*n* = 36), MRI scan (*n* = 11) or ^68^Ga-PSMA-PET/CT scan (*n* = 56). In retrospect, a prior ^68^Ga-PSMA-PET/CT scan led to the highest success rate of biopsies with sufficient tumor yield for molecular analysis (66.1%), compared to a prior CT or MRI scan (52.8% and 54.5%, respectively).

Our results indicate that fusion target planning and CBCT guidance are feasible alternatives to CT guidance for taking bone biopsies. Fusion imaging and CBCT are promising techniques to improve biopsy and ablation guidance [15, 16]. In this pilot study, in nine of ten (90%) biopsy cores PCa cells were found, and in eight patients (80%) molecular analysis using smMIP or WGS was successful. This suggests that high diffusion restriction and PSMA uptake are indicative for high tumor yield and that the planned needle path overlay assists in accurate needle placement. Due to the simultaneous introduction of the combination of ^68^Ga-PSMA-PET/CT and MRI as prior imaging modalities and a new innovative method with CBCT guidance to acquire tumor tissue, we were not able to differentiate which factor contributed most to the outcome of our pilot study. Further investigation is necessary to assess which planning image parameters best characterize the tumor through correlation with histology [17]. When the correlation between image parameters and tumor yield is better understood, a larger prospective study is required to reproduce the results and to compare CBCT guidance with CT-guided biopsies.

In conclusion, target planning on fused ^68^Ga-PSMA-PET/CT, MRI and CBCT image datasets and CBCT needle guidance were feasible and increased the success rate of molecular analysis on bone biopsies of mCRPC patients compared to the existing CT-guided biopsies studies as well as to our own historical cohort. The implementation of the biopsy procedure described in this study could improve precision medicine in patients with solely bone metastatic sites and for those with small bone metastatic lesions. In addition, the procedure could eventually be introduced earlier in the disease state with the potential of enhancing personalized treatment options and improving outcome for patients with metastatic prostate cancer.
